# Rapid Removal of Mercury from Water by Novel MOF/PP Hybrid Membrane

**DOI:** 10.3390/nano11102488

**Published:** 2021-09-24

**Authors:** Jian Gao, Ziming Li, Ziqi Deng, Meihua Liu, Wei Wei, Chunbai Zheng, Yifan Zhang, Shusen Chen, Pengyang Deng

**Affiliations:** 1 CAS Key Laboratory of High-Performance Synthetic Rubber and Its Composite Materials, Changchun Institute of Applied Chemistry, Chinese Academy of Sciences, Changchun 130022, China; jgao@ciac.ac.cn (J.G.); liumh@ciac.ac.cn (M.L.); weiwei@ciac.ac.cn (W.W.); zhengcb@ciac.ac.cn (C.Z.); pydeng@ciac.ac.cn (P.D.); 2 Beijing Research Institute of Chemical Engineering and Metallurgy, CNNC, Beijing 101149, China; liziming9209@126.com; 3 Department of Chemistry, College of Science, Yanbian University, Yanji 133002, China; bijingniqige@163.com; 4 University of Science and Technology of China, Hefei 230026, China

**Keywords:** metal–organic frameworks, membrane, mercury removal, rapid, regenerable

## Abstract

Mercury is one of the most toxic heavy metals that can cause terrible disease for human beings. Among different absorption materials, MOF (metal–organic framework) materials show potential as very attractive materials for the rapid removal of mercury. However, the instability and difficulty for regeneration of MOF crystals limit their applications. Here, a continuous sulfur-modified MOF (UiO-66-NHC(S)NHMe) layer was synthesized in situ on polymeric membranes (PP non-woven fabrics) by post-synthetic modification and used for rapid mercury removal. The MOF-based membrane (US-N) showed high selectivity for mercury in different aqueous systems, which is better than sulfur-modified MOF powders. A thinner MOF layer on US-N showed a much better mercury ion removal performance. US-N with a 59.3 nm MOF layer could remove more than 85% of mercury in 20 min from an aqueous solution. In addition, the US-N can simply regenerate several times for mercury removal and maintain the initial performance (removal ratio > 98%), exhibiting excellent durability and stability. This work promotes the application of MOF materials in the rapid removal of hazardous heavy metal ions from practical environments.

## 1. Introduction

Mercury, as one of the most toxic heavy metals, can accumulate in the human body through the food chain and lead to severe health issues [1,2]. Therefore, eliminating mercury from polluted water is of great importance in terms of the protection of human health [3]. In the past, various methods have been developed, for example, ion exchange, membrane filtration, chemical precipitation and adsorption. Among the above methods, adsorption is recognized as one of the most simple, efficient and economic methods for water decontamination applications [4,5]. A variety of adsorbents have been developed and tested for removing mercury from contaminated water. However, traditional adsorbents such as activated carbons [6], zeolites [7] and clays [8] usually suffer from low capacity, weak binding affinity and poor selectivity for mercury. Novel adsorbents with excellent mercury separation performance and high selectivity are highly pursued.

MOFs (metal–organic frameworks) are porous crystalline materials with high surface areas, tunable pore size and functional groups, exhibiting great advantages in the fields of catalysis, separation and adsorption [9,10,11]. Various functionalized MOF materials have been synthesized [12,13]; among them, thiol/thio-functionalized MOFs are considered one of the most effective adsorbents for mercury [14,15]. Thiol/thio-functionalized MOFs can remove mercury from aqueous solutions with a high removal ratio and high selectivity, resulting from the strong coordination between thiol and mercury [15,16,17,18] and the porous structures maximizing the accessibility of active sites [19]. For example, Mon and coworkers reported a bioMOF with thioether arms, which could selectively capture Hg(II) from water with a removal ratio of more than 99% [16]. He et al. combined thioether functions with Zr(IV)-based MOF materials, improving the mercury removal ratio to 96% [17]. Yang et al. constructed a kind of Zr-MSA MOF, the dense active alkyl thiols of which led to high adsorption capacity, ultra-fast adsorption kinetics and super-high capture efficiency for Hg^2+^ removal [18]. Although thiol/thio-functionalized MOFs show excellent mercury absorption performance, the instability of MOFs in water/aqueous solutions and the difficulty of recycling and regenerating MOF crystals are still challenges during application [20].

In this paper, sulfur-modified MOF (UiO-66-S) was synthesized in situ on polymeric membranes (UiO-66-NHC(S)NHMe@NWF-g-MAH, US-N) to improve the stability of MOFs. The selectivity for mercury, the adsorption kinetics and the regenerability of US-N were investigated. US-N showed high selectivity for mercury with excellent absorption and separation performance in different aqueous systems. The thinner MOF layer of US-N obtained faster separation speeds and a higher specific capacity of equilibrium absorption. Moreover, US-N can be easily regenerated and maintain a good removal ability. This work not only provides a promising high selective stable MOF membrane for the rapid removal of mercury, but also promotes the development of new membranes for the rapid removal of various hazardous heavy metal ions.

## 2. Materials and Methods

### 2.1. Chemicals and Materials

ZrCl_4_ (≥98.0%), 2-aminoterephthalic acid (≥98.0%), mercury standard solution (AAS, 1 mg/mL Hg in 2–5% HNO_3_) and lead standard solution (AAS, 1 mg/mL Pb in 2–5% HNO_3_) were purchased from Alfa Aesar. CH_3_NCS (≥99.0%) and hydroxyethyl starch (98%) were purchased from XiYa Chemical (Linyi, China) Technology. Zn(NO_3_)_2_·6H_2_O (≥98.0%), Cu(NO_3_)_2_·3H_2_O (≥98.0%), FeCl_3_·6H_2_O (≥98.0%), DMF (A.R.), acetone (A.R.) and C_2_H_5_OH (A.R.) were purchased from Aladdin Industrial Corporation (Shanghai, China). Tetrahydrofuran, K_2_Cr_2_O_7_ (A.R.), CdO (≥99.999%), Ni(NO_3_)_2_·6H_2_O (A.R.), CaCO_3_ (A.R.), MgSO_4_ (A.R.), Mn(NO_3_)_2_ (50%, A.R.), THF (A.R.) and HF (≥40%, A.R.) were purchased from Beijing Chemical Works (Beijing, China). Deionized water was used throughout all experiments. PTFE filters (13 mm diameter with pore size of 0.22 μm) were purchased from ZiYang Economic and Trade Corporation (Changchun, China). Substrates used in this work (non-woven fabric made of polypropylene (NWF) with 0.42 mm thickness and a mass density of 120 g/m^2^) were purchased from commercial sources. All substrates were washed with acetone and dried at 60 °C for 3 h in a vacuum oven before use. All reagents and materials were used as received without further treatment.

### 2.2. Synthesis-Modified MOF Membrane (US-N)

The preparation process of MOF membranes (UiO-66-NHC(S)NHMe@NWF-g-MAH, US-N) was as follows: 1.1 mmol ZrCl_4_ and 1.5 mmol ligand (BDC-NH_2_) were added to 15 mL DMF separately, sonicated until completely dissolved and then mixed together well. The NWF-g-MAH with 38 mm diameter was soaked in the mixed solution and positioned vertically in an autoclave; MOF membranes (UiO-66-NH_2_@NWF-g-MAH, UN-N) were synthesized in situ on the NWF-g-MAH at 120 °C with a reaction time of 24 h. After synthesis, the membranes were sonicated in DMF and ethanol several times separately, and immersed in hot ethanol for solvent exchange. Finally, the membranes were dried at 60 °C overnight.

Then, the UN-N membranes were suspended in a mixture of 1.5 mL MeOH and 13.5 mL CHCl_3_ and treated with 15 mg CH_3_NCS at 55 °C for 3 days. After completion of the reaction, the US-N was sonicated in CHCl_3_ to remove any by-products and soaked in fresh solvent for 24 h (3 times). Then, the US-N was dried at 60 °C for 12 h through vacuum filtration with a pressure of 100 mTorr.

### 2.3. Filter Removal Processes

US-N were activated at 120 °C for 2 h before being used for filtration. In a typical adsorption, US-N (3 pieces of membranes with 38 mm diameters) were added to a circulation device (Figure S5a,b) containing 200 mL solution of heavy metal ions (the pH was 2.6 ± 0.1), and the system was cycled for 2 h at room temperature. Finally, the residual metal content of the solution (the pH was 2.8 ± 0.2) was measured by ICP-MS.

Before the heavy metal removal experiment, all labware (necked bottle, Teflon tubes, funnel, fixtures and gasket) was boiled in nitric acid, rinsed with deionized water and dried to reduce adsorption of heavy metal ions.

### 2.4. Regeneration of US-N in Mercury Removal

After mercury adsorption, US-N membranes were immersed in 50 mL 0.1 M NH_4_Cl solution for 2 h, then rinsed with deionized water, dehydrated with ethanol and dried at 60 °C, and the US-N membranes were regenerated. The regenerated US-N membranes were applied for mercury removal again. The above process was repeated 9 times.

## 3. Results and Discussion

### 3.1. Synthesis and Characterization

**Scheme 1:**
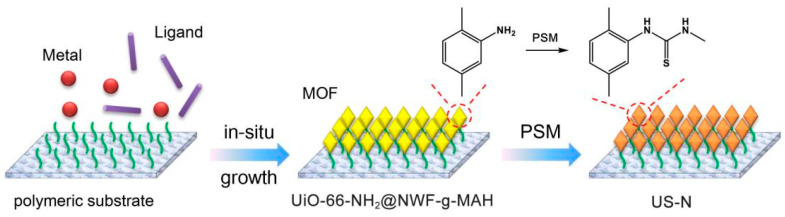
The preparation process diagram for US-Ns.

**Figure 1:**
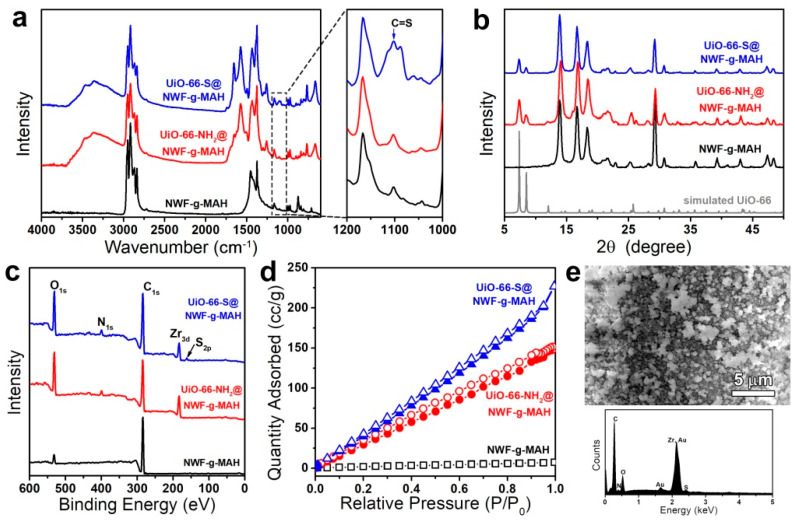
The characterization of US-Ns, (**a**) ATR-FTIR, (**b**) XRD, (**c**) XPS, (**d**) N_2_ isothermal adsorption curve, (**e**) SEM image and EDS spectrum; the scale bar is 5 μm.

The preparation process of US-N is illustrated in Scheme 1. First, the UiO-66-NH_2_ layer was solvothermally grown in situ on irradiation-treated polymeric substrate (non-woven fabrics), and then US-N were obtained after post-synthetic modification. The MOF membranes were characterized using Fourier transform infrared (FTIR, Vertex 70, Bruker, Karlsruhe, Germany) spectra, X-ray powder diffraction (XRPD, D8 Advance. Bruker) spectra, X-ray photoelectron spectroscopy (XPS, ESCALAB 250, Thermo Fisher Scientific, Waltham, MA, America), Brunauer–Emmett–Teller (BET, Quantachrome, Boynton Beach, FL, America) and a scanning electron microscope (SEM, XL-30, FEI, Hillsboro, OR, America) with energy dispersive spectrometer (EDS, Oxford X-MaxN 150, Oxford, Britain), as shown in Figure 1. As shown in Figure 1a, compared to the FTIR spectra of NWF-g-MAH and UiO-66-NH_2_@NWF-g-MAH, the new absorption peak at 1103 cm^−1^ was observed at US-N, which was attributed to the stretching mode of C=S [21]. In Figure 1b, the diffraction peaks of US-N at 7.36 and 8.48° matched with the (111) and (002) crystallographic planes of UiO-66 [22], which indicated that the UiO-66-S layer retained its original crystal structure and underlying topology on the substrate surface. In XPS spectra of US-N, the peak of sulfur appeared at 162 eV compared to that of NWF-g-MAH and UiO-66-NH_2_@NWF-g-MAH in Figure 1c, and the content of sulfur on US-N was 0.58 (At.%). The US-N exhibited a higher BET value (292.9 m^2^g^−1^) compared with UiO-66-NH_2_ membranes (224 m^2^g^−1^) measured using N_2_ adsorption–desorption isotherm (measured under 77 K) in Figure 1d. Figure 1e presents a continuous MOF layer with c.a. 220 nm particle size prepared on the substrate, and the content of S on US-N was 0.59 wt.% (Figure 1e).

### 3.2. Hg^2+^ Removal by US-N

**Figure 2:**
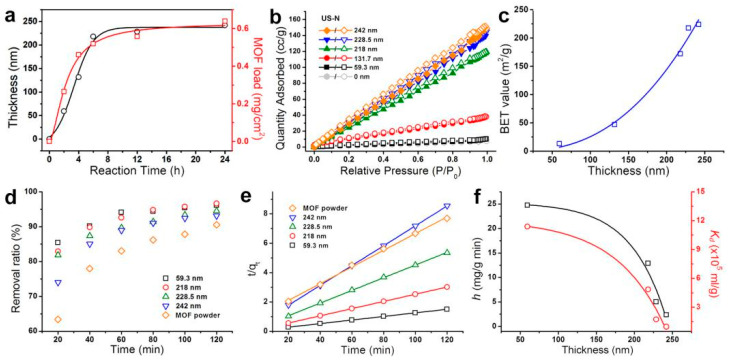
(**a**) MOF layer thickness and MOF load of US-N with different reaction times, (**b**) the nitrogen adsorption isotherms of US-Ns with different MOF layer thickness, (**c**) BET surface areas of US-Ns with different MOF layer thickness, (**d**) the Hg^2+^ removal performance of US-Ns with different MOF layer thickness, (**e**) pseudo-second-order kinetic models, (**f**) the initial sorption rate *h* (left, black) and the distribution ratio *K_d_* (right, red) versus layer thickness.

To investigate the Hg^2+^ removal performance of US-N, US-Ns with different layer thicknesses of MOF were prepared by tuning the solvothermal reaction time. The thickness of MOF layers prepared at 2 h, 4 h, 6 h, 12 h and 24 h were 59.3 nm, 131.7 nm, 218.0 nm, 228.5 nm and 242.0 nm, respectively, as shown in Figure 2a (details in Table S2 and Figure S3). The MOF load of US-N prepared at 2 h, 4 h, 6 h, 12 h and 24 h were 0.265 mg/cm^2^, 0.461 mg/cm^2^, 0.519 mg/cm^2^, 0.558 mg/cm^2^ and 0.640 mg/cm^2^, respectively, which were calculated from ICP-MS results. Nitrogen adsorption isotherms of US-N with different thicknesses were measured and the BET was calculated, as shown in Figure 2b,c. It was found that the BET value of US-N increased from 13 to 224 m^2^/g as the thickness of the MOF layer increased from 59.3 nm to 242 nm, indicating the adsorption surface area of US-N increases with the thickness of the MOF layer.

The Hg^2+^ removal ratios of different US-Ns were measured by detecting the concentration of Hg^2+^ solution in a circulation filtration device (Figure S5) before and after adding US-N for certain times. Compared with UiO-66-S powder (90.5% removal ratio for Hg^2+^ after 120 min adsorption), the US-N with different layer thicknesses of MOF all exhibited a better Hg^2+^ removal performance, as shown in Figure 2d. To our surprise, the Hg^2+^ removal ratio increased significantly with the layer thickness decreasing from 242.0 to 59.3 nm. For the US-N with a MOF layer thickness of 59.3 nm, the Hg^2+^ removal ratio after 20 min filtration reached 85%, whilst after 120 min, it exceeded 96%, exhibiting a high purification ability.

The pseudo-second-order model is applied to analyze the adsorption process of US-N in solution, which is expressed as:

(1) $$\frac{t}{q_{t}}=\frac{1}{k_{2}{q_{e}}^{2}}+\frac{t}{q_{e}}$$

where *k*_2_ is the rate constant of pseudo-second-order adsorption (g∙mg^−1^∙min^−1^), *q_t_* (mg∙g^−1^) is the amount of Hg^2+^ adsorbed at time *t* (min) and *q_e_* is the amount of Hg^2+^ adsorbed at equilibrium (mg∙g^−1^). The slope and intercept of the linear plot *t*/*q_t_* versus *t* yield the values of *q_e_* and *k*_2_. *h* can be regarded as the initial sorption rate, *q_t_*/*t*, when *t* approaches 0:

(2) $$h=k_{2}{q_{e}}^{2}$$

The distribution ratio *K_d_* (mL/g) was calculated to analyze the scavenging performance of the US-N:

(3) $$K_{d}=\frac{C_{0}-C_{e}}{C_{e}}\times \frac{V}{m}$$

where *C*_0_ and *C_e_* denote the initial and equilibrium concentrations of metal ions in the aqueous phase, respectively, *V* is the volume of the treated solution (ml) and *m* is the weight of adsorbent used (g).

Hg^2+^ removal of US-N with different layer thicknesses is consistent with the pseudo-second-order model (R-square > 0.99), as shown in Figure 2e. Figure 2f shows that *h* increased when the layer thickness of US-N decreased (or as the reaction time decreased), demonstrating that the thinner the MOF layer thickness, the faster the removal rate of mercury ions. The distribution coefficient *K_d_* values of all US-N were higher than that of US powder (approximately 10^5^ mL/g, as shown in Table S4), and *K_d_* increased as the layer thickness decreased, as shown in Figure 2f. For US-N with 59.3 nm (2 h), *K_d_* reached 11.38 × 10^5^ mL/g, indicating the considerably improved scavenging performance of US-N with thinner MOF layers.

The above results demonstrate that under the premise of uniform and complete coverage of the scaffold by MOF layer, the thinner the US-N, the better scavenging performance of Hg^2+^. Compared with UiO-66-S powder, the removal efficiency of US-Ns is notably superior. This improvement is attributed to the smaller size of MOF particles on our membranes, resulting in an increased percentage of accessible functional groups on the surface materials with the decrease in its size. The surface-exposed functional groups are active, and consequently, US-N with smaller MOFs exhibit higher chemical activity than larger, loose MOF powders.

### 3.3. Selectivity of US-N to Hg^2+^

**Figure 3:**
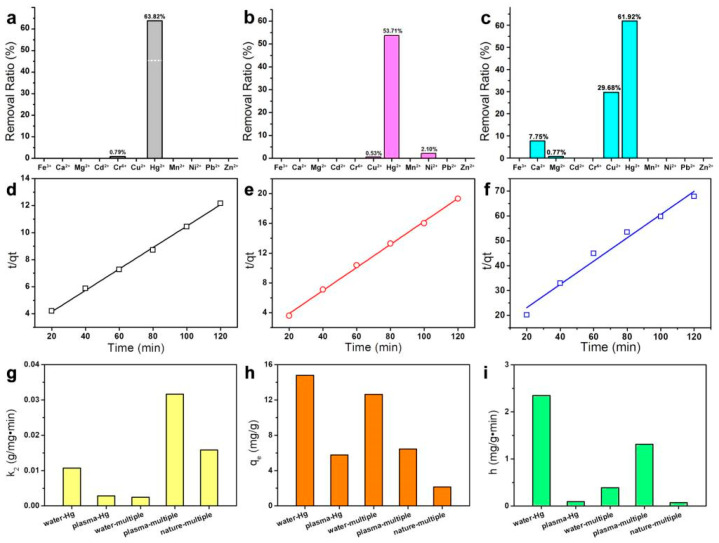
The selectivity for US-N for the removal of 11 multiple metals in (**a**) pure water, (**b**) artificial plasma and (**c**) natural water. Pseudo-second-order kinetic models for US-N to mercury in the removal of 11 multiple metal ions, (**d**) in water, (**e**) in artificial plasma and (**f**) in natural water. The fitting parameters for US-N to Hg^2+^ in different systems, Hg^2+^ in water, Hg^2+^ in artificial plasma, multiple metal ions in water, multiple metal ions in artificial plasma, and multiple metal ions in natural water, (**g**) adsorption rate constant *k*_2_, (**h**) equilibrium adsorption *q_e_* and (**i**) initial sorption rate *h*.

The selectivity of US-N was investigated by measuring the removal ratio of 11 kinds of metal ions in different systems, including a pure water system, an artificial plasma system and a natural water system (for details, see Supplementary Materials Section S3.4). The removal ratio of 11 metal ions by US-N in water is shown in Figure 3a. The US-N exhibited excellent selectivity for Hg^2+^, and the removal ratio was up to 63.82%, compared to the removal ratio 45.45% of Hg^2+^ by US powder (Figure S6). In contrast, the removal ratio for Cr^6+^ was 0.79%, and for the other nine metals, almost zero (for details, see Table S8). This was attributed to the synergistic effect of porous structures and the specific binding ability of sulfur atoms to mercury, showing obvious advantages in multiple ions competition. Similar results can be observed in artificial plasma systems, shown in Figure 3b. The removal ratio for Hg^2+^ in artificial plasma was 53.71%. While the removal ratio of Cu^2+^ and Ni^2+^ was 0.53% and 2.10%, the removal ratio of other metal ions (Fe^3+^, Ca^2+^, Mg^2+^, Cd^2+^, Cr^6+^, Mn^2+^, Pb^2+^ and Zn^2+^) was almost zero. The removal ratio of Hg^2+^ in an artificial plasma system was lower than that in water; this was possibly caused by the fact that the viscosity of artificial plasma (4.10 × 10^−3^ Pa∙s, 30 °C) is much larger than water (0.8949 × 10^−3^ Pa∙s, 25 °C), and the mass transfer rate of heavy metal ions in solution decreases. In addition, heavy metal removal in natural water was studied, with the results shown in Figure 3c. It can be noted that the removal ratio of Hg^2+^ in natural water was 61.92%, which is similar to that in pure water, demonstrating that US-N can be applied in mercury removal from true environments.

US-N showed excellent selectivity for mercury. The removal kinetics of Hg^2+^ in the above systems was also investigated, as shown in Figure 3d–f. The Hg^2+^ removal performance of US-N in pure water, artificial plasma and natural water are all consistent with pseudo-second-order models. The adsorption rate constant (*k*_2_), equilibrium adsorption (*q_e_*) and initial sorption rate (*h*) in the four systems (Water-Multiple: aqueous solution with multiple metal ions including Fe^3+^, Ca^2+^, Mg^2+^, Cd^2+^, Cr^6+^, Cu^2+^, Hg^2+^, Mn^2+^, Ni^2+^, Pb^2+^ and Zn^2+^; Plasma-Multiple: artificial plasma system with multiple metal ions including Fe^3+^, Ca^2+^, Mg^2+^, Cd^2+^, Cr^6+^, Cu^2+^, Hg^2+^, Mn^2+^, Ni^2+^, Pb^2+^ and Zn^2+^; Water-Hg: aqueous solution of Hg^2+^; Plasma-Hg: artificial plasma system with Hg^2+^) were compared in Figure 3g–i (for details, see Table S10). It could be easily found that: (1) the highest adsorption rate constant was obtained in the plasma-multiple (Figure 3g), and this was attributed to the low equilibrium adsorption and the high initial sorption rate; (2) the highest equilibrium adsorption was obtained in water-Hg, and decreased significantly in the plasma system (Figure 3h); and (3) the highest initial sorption rate was obtained in water-Hg, but the plasma-multiple was higher than the water-multiple (Figure 3i), and the different trend in *q_e_* and *h* led to the result of *k*_2_.

### 3.4. Regeneration of US-N in Mercury Removal

**Figure 4:**
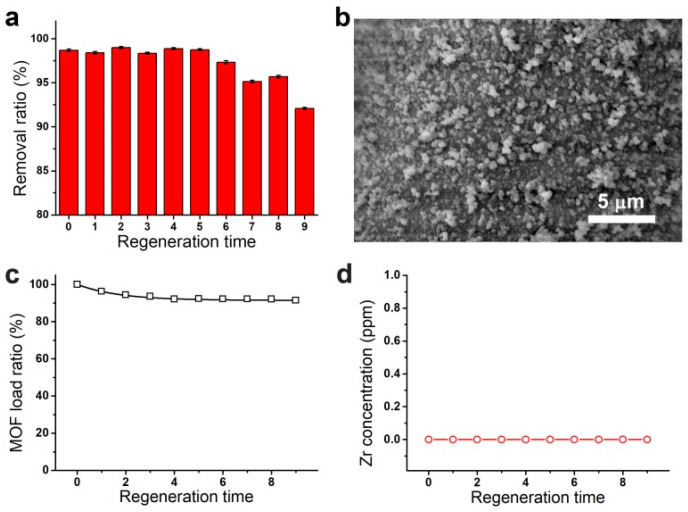
Mercury adsorption after repeated regenerations. (**a**) Removal ratio of Hg^2+^ by US-N, (**b**) SEM image of US-N after 9 regenerations, (**c**) the remaining MOF load ratio on US-N after different regeneration cycles, (**d**) the Zr^4+^ concentration in filtrate after different regeneration cycles.

To study the regeneration of US-Ns, the US-N membranes were regenerated after mercury ion adsorption was completed nine times, and the adsorption performance of the regenerated membranes was tested (details in Supplementary Materials Section S3.7). The removal ratio of mercury ions in different regeneration times is shown in Figure 4a and Table S11. The original removal ratio of mercury for US-N was 98.68%, and the removal ratios in five regeneration times were all above 98%. After the fifth regeneration time, the removal ratio slightly decreased, and at the ninth regeneration time, the adsorption performance decreased to 92%. The surface morphology of US-N after regeneration was observed, as shown in Figure 4b, and no obvious cracks or defects were observed on the samples. The MOF load was also detected. The ratio of remaining MOFs on US-N after each regeneration cycle, compared to the original US-N, is shown in Figure 4c. The ratio only slightly decreased to 92% after nine regenerations. The decrease is possibly due to the MOF particles that weakly adhered to the membrane falling off during regeneration cycles. Moreover, no Zr^4+^ was detected in filtrate during the nine regeneration cycles (Figure 4d), which means that no MOF particle dropped from the membrane in the removal process.

The results show that the US-N could be easily regenerated after adsorption, and the regenerated US-N maintained their high performance and their morphology. The US-N is durable during regeneration cycles, showing only 8% loss of MOF after nine regenerations. This capability is highly desired for industrial applications.

## 4. Conclusions

In summary, a sulfur-modified MOF membrane (US-N) was prepared and applied in heavy metal removal. In the mixed solution of 11 metal ions, US-N showed a high selectivity for mercury, regardless of whether in water, artificial plasma or natural water. The removal kinetics of mercury showed that the removal process fits pseudo-second-order kinetic models, and a thinner MOF layer on US-N showed much faster separation speeds and a higher specific capacity of equilibrium absorption. Moreover, this MOF membrane can simply be regenerated after mercury ion removal, and maintain its initial performance. Our work presents a potential industrial application for MOF-based membranes.

## Data Availability

Not applicable.

## Supplementary Materials

The following are available online at https://www.mdpi.com/article/10.3390/nano11102488/s1, Figure S1: Calibration curve of MOF weight versus zirconium concentration for the calculation of MOF weight, Figure S2: The characterization of NWF-g-MAH, Figure S3: The cross-section SEM images of US-N with different reaction time (2–24 h) and the relationship between MOF load and reaction time, Figure S4: The SEM images of US-N with different reaction times and the relationship between MOF particle size and reaction time, Figure S5: Schematic and photo image of the filtering circulation device, Figure S6: The removal ratio for 11 metals for US-N and US powder, Figure S7: Removal ratio and pseudo-second-order kinetic models for US-N to mercury in the removal of 11 multiple metals; Figure S8: Removal ratio and pseudo-second-order kinetic models for US-N to mercury in single mercury removal, Table S1: UiO-66-NHC(S)NHMe weights and zirconium concentrations for the calibration curve, Table S2: The details for US-N prepared by different reaction times, Table S3: The details for US-N prepared by different reaction times, Table S4: Coefficients of pseudo-second-order models for MOF membranes with different reaction times and MOF powder, Table S5: The target concentration and stock solution volume for 11 metals added in the mixed multi-metals solution, Table S6: The target concentration and metal added in the mixed multi-metals solution, Table S7: The original concentration for 11 heavy metals in natural water, Table S8: The removal ratio for 11 metals in different solutions cycled after 2 h, Table S9: The removal ratio of Hg under different concentrations, Table S10: The pseudo-second-order kinetic parameters for mercury removal in different situations, Table S11: Mercury adsorption removal ratio after repeated regeneration.
