# Supplementary material for: Rapid Removal of Mercury from Water by Novel MOF/PP Hybrid Membrane
Source: Nanomaterials (Basel). 2021 Sep 24;11(10):2488. doi: 10.3390/nano11102488 (PMC8539959; doi:10.3390/nano11102488)
Supplement: Supplementary file 1 [file nanomaterials-11-02488-s001.zip › nanomaterials-1363104-supplementary.pdf]

Supplementary Materials

Rapid Removal of Mercury from Water by Novel MOF/PP Hybrid Membrane

Jian Gao [1,†], Ziming Li [2,†], Ziqi Deng [3], Meihua Liu [1], Wei Wei [1], Chunbai Zheng [1], Yifan Zhang [1,*], Shusen Chen [2,*] and Pengyang Deng [1,4]

1 CAS Key Laboratory of High-Performance Synthetic Rubber and Its Composite Materials, Changchun Institute of Applied Chemistry, Chinese Academy of Sciences, Changchun 130022, China; jgao@ciac.ac.cn (J.G.); liumh@ciac.ac.cn (M.L.); weiwei@ciac.ac.cn (W.W.); zhengcb@ciac.ac.cn (C.Z.); pydeng@ciac.ac.cn (P.D.)

2 Beijing Research Institute of Chemical Engineering and Metallurgy, CNNC, Beijing 101149, China; liziming9209@126.com

3 Department of Chemistry, College of Science, Yanbian University, Yanji 133002, China; bijingniqige@163.com (Z.D.)

4 University of Science and Technology of China, Hefei 230026, China

* Correspondence: yfzhang@ciac.ac.cn (Y.Z.); samcss@163.com (S.C.); Tel.: 86-0431-85262329 (Y.Z)

† These authors contributed equally to this work.

1. Materials

$ZrCl_4$ (≥98.0%), 2-aminoterephthalic acid (≥98.0%), mercury standard solution (AAS, 1 mg/ml Hg in 2-5% HNO_3) and lead standard solution (AAS, 1 mg/ml Pb in 2-5% HNO_3) were purchased from Alfa Aesar. CH_3NCS (≥99.0%) and hydroxyethyl starch (98%) were purchased from XiYa Chemical Technology. $Zn(NO_3)_2{\cdot}6H_2O$ (≥98.0%), $Cu(NO_3)_2{\cdot}3H_2O$ (≥98.0%), $FeCl_3{\cdot}6H_2O$ (≥98.0%), DMF (A.R.), acetone (A.R.) and C_2H_5OH (A.R.) were purchased from Aladdin Industrial Corporation. Tetrahydrofuran, $K_2Cr_2O_7$ (A.R.), CdO (≥99.999%), $Ni(NO_3)_2{\cdot}6H_2O$ (A.R.), $CaCO_3$ (A.R.), $MgSO_4$ (A.R.), $Mn(NO_3)_2$ (50%, A.R.), THF (A.R.), and HF (≥40%, A.R.) were purchased from Beijing Chemical Works. Deionized water was used throughout all experiments.

PTFE filter (13 mm diameter with pore size of 0.22 μm) was purchased from ZiYang Economic and Trade Corporation.

Substrates used in this work were purchased from commercial sources, and detail is: non-woven fabric made of polypropylene (NWF) with 0.42 mm thickness and the mass density is 120 g/m^2.

All substrates were washed by acetone and dried at 60°C for 3 h in a vacuum oven before use.

All reagents and materials in this work were used as received without further treatment.

2. Characterization

2.1. Measurement of Hg^{2+} concentration in aqueous solution

The Hg^{2+} concentration (mg/L, ppm) in aqueous solution was measured by ICP-MS (ThermoScientific X-series^{II}, USA). To prevent hydration of Hg^{2+} at low concentrations, 30 μL of 1 wt.% $K_2Cr_2O_7$ was added to the liquid sample for measurement (using the formulation 0.01 wt.% $K_2Cr_2O_7$ and 5% HNO_3) to maintain the concentration of Hg^{2+}.[1]

The adsorption kinetics of Hg^{2+} by MOF membranes were investigated with the pseudo-second-order model, which is expressed as:

$$\frac{t}{q_t} = \frac{1}{k_2 {q_e}^2} + \frac{t}{q_e} \tag{1}$$

where k_2 is the rate constant of pseudo-second-order adsorption (g·mg^{-1}·min^{-1}), q_t (mg·g^{-1}) is the amount of Hg^{2+} adsorbed at time t (min) and q_e is the amount of Hg^{2+} adsorbed at equilibrium (mg·g^{-1}). The slope and intercept of the linear plot t/q_t versus t yield

the values of q_e and k_2. h can be regarded as the initial sorption rate, q_t/t, when t approaches 0:

$$h = k_2 q_e^{\,2} \tag{2}$$

The distribution ratio K_d (ml/g) was calculated to analyse the scavenging performance of the US-N:

$$K_d = \frac{C_0 - C_e}{C_e} \times \frac{V}{m} \tag{3}$$

where C_0 and C_e denote the initial and equilibrium concentrations of metal ions in the aqueous phase, respectively, V is the volume of the treated solution (ml), and m is the weight of adsorbent used (g).

2.2. Measurement of metal-organic framework (MOF) loading

The MOF loading on the MOF membrane was measured by inductively coupled plasma-mass spectrometry (ICP-MS, ThermoScientific X-series^{II}, USA) by using the following procedures:

(i) Calibration curve preparation: UiO-66-NHC(S)NHMe powder was activated at 120°C for 2 h under vacuum, weighed and divided into five parts with different mass (Table S1). The powders were mixed with 800 μl HF and heated at 55°C for 30 min for complete degradation, then rinsed repeatedly with deionized water. All the liquid was transferred to a 100 ml volumetric flask and mixed well. Then, 3 ml of solution was removed to measure the Zr ion concentration by ICP-MS. The concentration of zirconium ions increased as the weight of MOF powder increased. The calibration curve of MOF weight (M_{MOF}, mg) *versus* zirconium concentration (C_{Zr}, ppm) was thus obtained (Table S1, Figure S1, R-square = 0.99548) and is expressed as equation (4), where 100 ml is converted to 0.1 L.

(ii) Calculation of MOF loading: The MOF membrane (UiO-66-NHC(S)NHMe@NWF-g-MAH, US-N) was treated using the same process as above. The concentration of zirconium was obtained after the test by entering M_{MOF} (mg) into equation (4). The MOF loading (mg/cm^2) could thus be calculated using equation (5), in which $S_{substrate}$ (cm^2) is the surface area of the substrate (double-sided for films).

$$C_{Zr} = \frac{0.02903 + 0.248873 \times M_{MOF}}{0.1} \tag{4}$$

$$M_{MOF} = \frac{C_{Zr} - 0.2903}{2.48873} \tag{5}$$

Table S1. UiO-66-NHC(S)NHMe weights and zirconium concentrations for the calibration curve.

Sample	MOF weight (mg)	Zr concentration (ppm, mg/L)
Powder 1	13.7	36.38
Powder 2	18.1	41.72
Powder 3	20.4	53.09
Powder 4	39	98.16
Powder 5	43.4	105.6
Powder 6	55.5	139.9

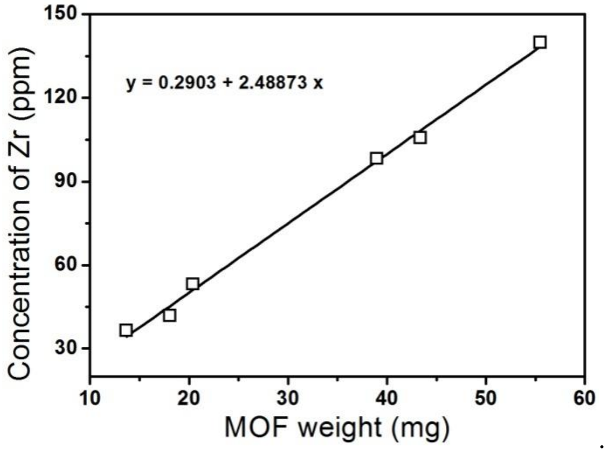

Figure S1. Calibration curve of MOF weight *versus* zirconium concentration for the calculation of MOF weight.

3. Results

3.1. Chacteriazation of NWF-g-MAH

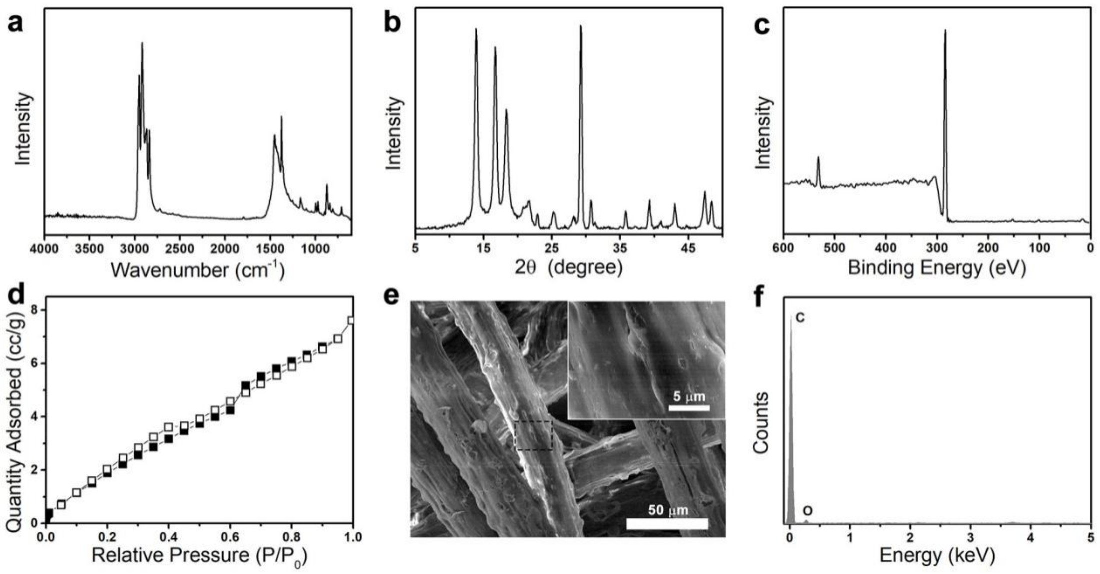

Figure S2. The characterization of NWF-g-MAH, a) ATR-FTIR, b) XRD, c) XPS, d) N2 isothermal adsorption curve, e) SEM image and f) EDS spectrum.

3.2. Synthesis modified MOF membrane (US-N)

The preparation of modified MOF powder (UiO-66-NHC(S)NHMe, US) and membrane (UiO-66-NHC(S)NHMe@NWF-g-MAH, US-N) was same as previously report,[2,3] the general process as followed:

Amount of $ZrCl_4$ and ligand (BDC-NH_2) were added to DMF, sonicated for completely dissolved separately and mixed together well. The NWF-g-MAH with 38 mm diameter was soaked in the mixed solution and positioned vertically in autoclave; MOF membranes (UiO-66-NH_2@NWF-g-MAH, UN-N) were in situ synthesized on the NWF-g-MAH at 120 °C with a reaction time of 24 h. After synthesis, the membranes were sonicated in DMF and ethanol several times separately, and immersed in hot ethanol for solvent exchange. Finally, dried at 60 °C overnight.

Then the UN-N membranes were suspended in a mixture of 1.5 ml MeOH and 13.5 ml $CHCl_3$ and treated with 15 mg CH_3NCS at 55°C for 3 days. After completion of the reaction, the US-N was sonicated in $CHCl_3$ to remove any by-products and soaked in fresh

solvent for 24 h (3 times). Then, the US-N was dried at 60°C for 12 h through vacuum filtration with a pressure of 100 mTorr.

3.3. Effect of MOF layer thickness of US-N on the mercury removal

3.3.1. MOF membranes with different layer thicknesses

The layer thickness of US-N prepared by the solvothermal method was tuned by the reaction time. The preparation of UN-N with different reaction times followed the process depicted in Section 3.1. The thicknesses of UN-N prepared at 2 h, 4 h, 6 h, 12 h and 24 h were 59.3 nm, 131.7 nm, 218.0 nm, 228.5 nm and 242.0 nm, respectively. And the load rate of UN-N are from 8-10%. The nitrogen adsorption isotherms of MOF membranes with different thicknesses were measured, and the BET surface areas of UN-N were calculated, as shown in Table S2. The BET surface area of the UN-N increases from 13 to 224 m^2/g as the thickness of MOF layer rises from 59.3 nm to 242 nm, indicating that the surface area of UN-N increases with MOF layer thickness.

After preparation, the UN-N with different thicknesses was modified to yield US-N, the modification process followed that described in Section 3.2. The modification do not change the thickness of MOF layer. The MOF loadings of US-N prepared at 2 h, 4 h, 6 h, 12 h and 24 h were 0.265 mg/cm^2, 0.558 mg/cm^2, 0.461 mg/cm^2, 0.519 mg/cm^2 and 0.640 mg/cm^2, respectively, as calculated from ICP-MS results.

Table S2. The details for UN-N prepared by different reaction time.

Reaction time	Thickness (nm)	BET (m^2/g)
2 h	59.3	13.14
4 h	131.7	47.03
6 h	218.0	171.97
12 h	228.5	217.73
24 h	242.0	223.95

Table S3. The details for US-N prepared by different reaction time.

Reaction time	Particle size (nm)	Load (mg/cm^2)
2 h	94.5	0.265
4 h	164.4	0.461
6 h	199.62	0.519
12 h	218.66	0.558
24 h	229.97	0.640

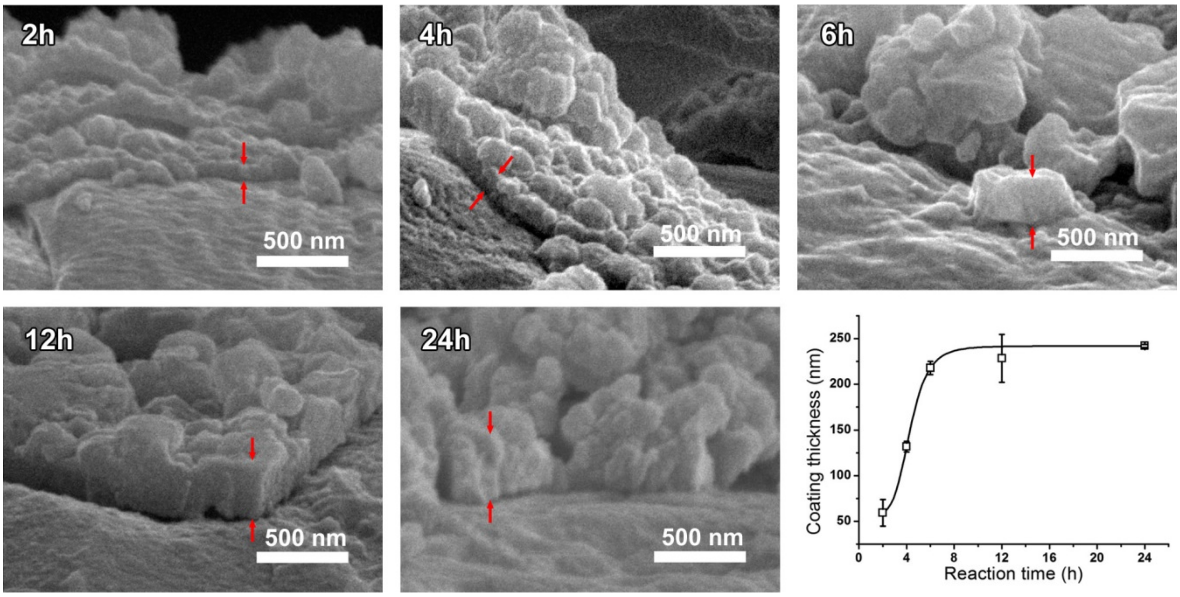

Figure S3. The cross-section SEM images of US-N with different reaction time (2-24 h) and the relationship between MOF coating thickness and reaction time. The scale bar is 500 nm, the red arrow was the measured place marked.

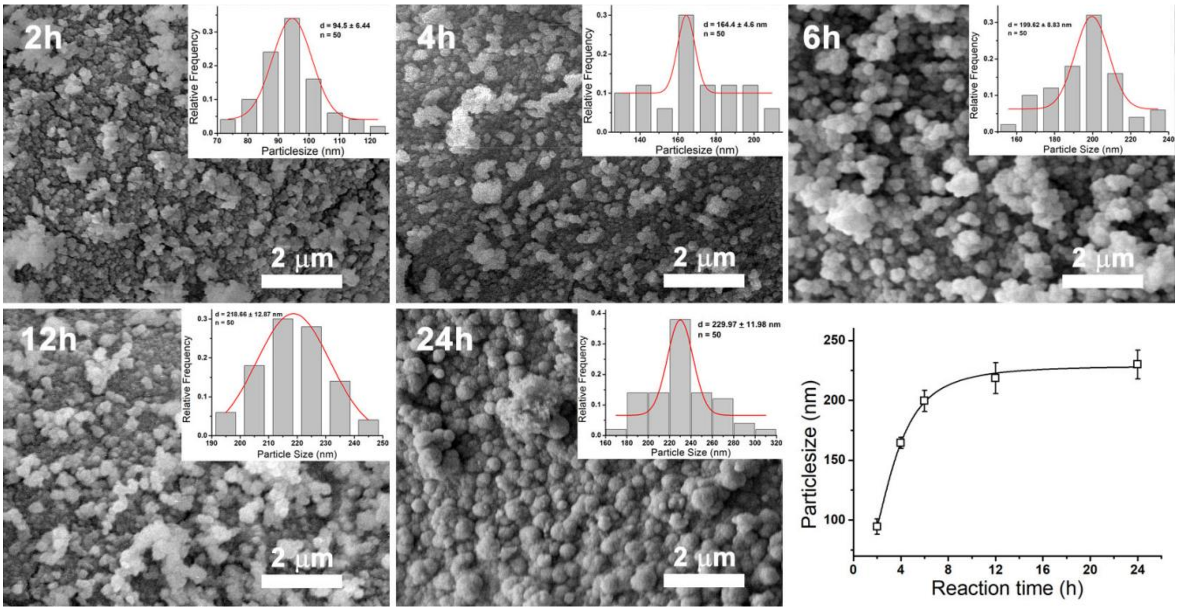

Figure S4. The SEM images of US-N with different reaction time and the relationship between MOF particle size and reaction time.

3.3.2. Filter removal processes

In a typical adsorption, US-N (3 pieces of membranes with 38 mm diameter) were added to a circulation device (Figure S5a and b) containing 200 ml of solution of heavy metal ions, and the system was cycled for 2 h at room temperature. Finally, the residual metal content of the solution was measured by ICP-MS.

Before the heavy metal removal experiment, all labwares (necked bottle, Teflon tubes, funnel, fixtures and gasket) were boiled in nitric acid, rinsed with deionized water and dried to reduce adsorption of heavy metal ions.

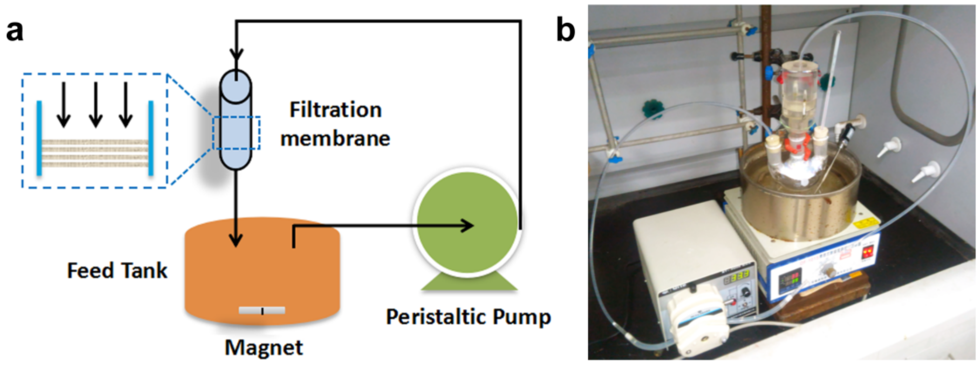

Figure S5. **(a)** Schematic and **(b)** photo image of the filtering circulation device.

3.3.3. Mercury removal by US-N with different layer thickness

Aqueous mercury solutions with a concentration of 2 ppm were prepared by diluting a standard solution with an appropriate amount of distilled water. The concentrations of Hg^{2+} during the experiments were determined by ICP-MS.

The Hg^{2+} removal experiment for US-N was preceded as depicted in Section 3.3.2, the initial concentration of Hg^{2+} is 2 ppm. And for comparison, another 200 ml aqueous solution of Hg^{2+} with a concentration of 2 ppm was mixed with 19.0 mg MOF powder adsorbent (UiO-66-NHC(S)NHMe) and stirred at room temperature for 2 h. During the adsorption period, the mixture was filtered at intervals through a 0.22 μm membrane filter, and then the filtrate was measured by using ICP-MS to determine the residual metal content.

Table S4. Coefficients of pseudo-second-order models for MOF membranes with different reaction time and MOF powder.

Sample	Thickness (nm)	R^2	k_2 (×10^{-3} g/mg ·min)	q_e	h	K_d (×10^5 ml/g)
2 h	59.3	0.99991	3.677	82.034	24.746	11.3815
6 h	218.0	0.99997	7.735	40.783	12.865	4.8623
12 h	228.5	0.99961	9.348	23.164	5.016	1.7362
24 h	242.0	0.99998	10.733	14.791	2.348	0.9704
powder		0.99773	3.297	17.609	1.022	1.0070

The adsorption of glass surface (the filtering circulation device without US-N) was also measured as a comparison, the process is the same as that depicted in Section 3.3.2. The adsorption ratio of Hg by the filtering circulation device is 1.62 ± 0.52 %.

3.4. Multiple metal solution removal

3.4.1. Multiple metals solution preparation

Stock metal solution preparation

Eleven metals (Fe^{3+}, Ca^{2+}, Mg^{2+}, Cd^{2+}, Cr^{6+}, Cu^{2+}, Hg^{2+}, Mn^{2+}, Ni^{2+}, Pb^{2+} and Zn^{2+}) were selected as target ions. Stock solutions of heavy metal ions with 1000 ppm concentrations were prepared by dissolving the relevant metal salts in water as follows.

Ca^{2+} solution: 0.2771 g $CaCl_2$ was added to a 100 ml volumetric flask and diluted with deionized water.

Mg^{2+} solution: 0.4951 g $MgSO_4$ was added to a 100 ml volumetric flask and diluted with deionized water.

Cd^{2+} solution: 0.1142 g CdO was added to a bottle, 2 ml 50% (v) HCl solution was added, and the mixture was sonicated to dissolve the salt. This solution was added to a 100 ml volumetric flask, diluted with deionized water and shaken well.

Cr^{6+} solution: 0.2829 g $K_2Cr_2O_7$ was added to a 100 ml volumetric flask and diluted with deionized water.

Cu^{2+} solution: 0.3802 g $Cu(NO_3)_2{\cdot}3H_2O$ was added to a 100 ml volumetric flask and diluted with deionized water.

Mn^{2+} solution: 651.5 μl $Mn(NO_3)_2$ solution (50%) was added to a 100 ml volumetric flask and diluted with deionized water.

Ni^{2+} solution: 0.4955 g $Ni(NO_3)_2{\cdot}6H_2O$ was added to a 100 ml volumetric flask and diluted with deionized water.

Zn^{2+} solution: 0.4548 g $Zn(NO_3)_2{\cdot}6H_2O$ was added to a 100 ml volumetric flask and diluted with deionized water.

Hg^{2+} and Pb^{2+} stock solution were commercial standard solution purchased.

Multiple metal ions in water preparation

Multipl metals solution with different initial metal concentration, (Fe^{3+} is 400 ppm, Ca^{2+} is 50 ppm, Mg^{2+} is 40 ppm, and Cd^{2+}, Cr^{6+}, Cu^{2+}, Hg^{2+}, Mn^{2+}, Ni^{2+}, Pb^{2+}, Zn^{2+} are 2 ppm), were prepared by mixing the relevant metal stock solution (as Table S5 showed) and diluted with deionized water in a 250 ml volumetric flask.

Table S5. The target concentration and stock solution volume for 11 metal ions added in the mixed multi heavy metals solution.

Metal	Fe^{3+}	Ca^{2+}	Mg^{2+}	Cd^{2+}	Cr^{6+}	Cu^{2+}	Hg^{2+}	Mn^{2+}	Ni^{2+}	Pb^{2+}	Zn^{2+}
Concentration (ppm)	400	50	40	2	2	2	2	2	2	2	2
Stock Solution volume (ml)	100	12.5	10	0.5	0.5	0.5	0.5	0.5	0.5	0.5	0.5

Multiple metal ions in artificial plasma preparation

60.0 g hydroxyethyl starch (HES) and 9.0 g NaCl were added into a 1 L volumetric flask and diluted with deionized water, ultrasonic first and magnetic stirred over night for dissolve completely.

Multi heavy metals solution in artificial plasma with different initial metal concentration, (Fe^{3+} is 400 ppm, Ca^{2+} is 50 ppm), Mg^{2+} is 40 ppm, and Cd^{2+}, Cr^{6+}, Cu^{2+}, Hg^{2+}, Mn^{2+}, Ni^{2+}, Pb^{2+}, Zn^{2+} are 2 ppm), were prepared by mixing the relevant metal salts or stock solution (as Table S6 showed) and diluted with artificial plasma prepared above in a 250 ml volumetric flask.

Table S6. The target concentration and metal added in the mixed multiple metals solution.

Metal	Fe^{3+}	Ca^{2+}	Mg^{2+}	Cd^{2+}	Cr^{6+}	Cu^{2+}	Hg^{2+}	Mn^{2+}	Ni^{2+}	Pb^{2+}	Zn^{2+}
Concentration (ppm)	400	50	40	2	2	2	2	2	2	2	2
Metal added (g or ml)	0.4841 g	0.0312 g	0.0495 g	0.5	0.5	0.5	0.5	0.5	0.5	0.5	0.5

Mercury in natural water

Natural water was obtained from Nanhu Park, and flited by 0.22 μm filter. Then 0.4 ml of mercury standard solution (1000 ppm) was added to a three-necked flask, diluted with 200 ml natural water and cycled for constant temperature without MOF membrane filter.

Table S7. The original concentration for 11 metal ios in natural water.

Metal	Fe^{3+}	Ca^{2+}	Mg^{2+}	Cd^{2+}	Cr^{6+}	Cu^{2+}	Hg^{2+}	Mn^{2+}	Ni^{2+}	Pb^{2+}	Zn^{2+}
Concentration (ppb)	57.13	54970	10350	0.425	0	5.533	1218	9.463	0	1.314	9.354

3.4.2. Single mercury solution preparation

Single mercury solution preparation

0.4 ml of mercury standard solution (1000 ppm) was added to a three-necked flask, diluted with 200 ml deionized water and cycled for constant temperature without MOF membrane filter.

Single mercury in artificial plasma preparation

0.4 ml of mercury standard solution (1000 ppm) was added to a three-necked flask, diluted with 200 ml artificial plasma prepared above and cycled for constant temperature without MOF membrane filter.

3.4.3. Multiple metal ions removal

3.4.3.1. Multiple Metals Solution

Table S8. The removal ratio for 11 metal ions in different solution cycled after 2 h.

**Removal ratio (%)**	**Fe^{3+}**	**Ca^{2+}**	**Mg^{2+}**	**Cd^{2+}**	**Cr^{6+}**	**Cu^{2+}**	**Hg^{2+}**	**Mn^{2+}**	**Ni^{2+}**	**Pb^{2+}**	**Zn^{2+}**
Water	0	0	0	0	0.79	0	63.82	0	0	0	0
Artificial Plasma	0	0	0	0	0	0.53	53.71	0	2.10	0	0
Natural Water	0	7.75	0.77	0	0	29.68	61.92	0	0	0	0
MOF powder in water	0	0	0	0	0	0	45.45	0	0	0	0

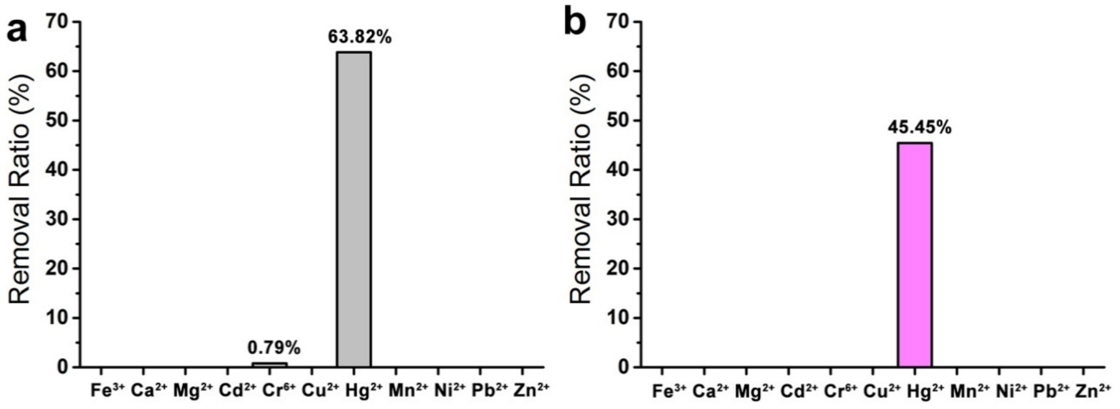

Figure S6. The removal ratio for 11 metal ions for US-N (a) and US powder (b).

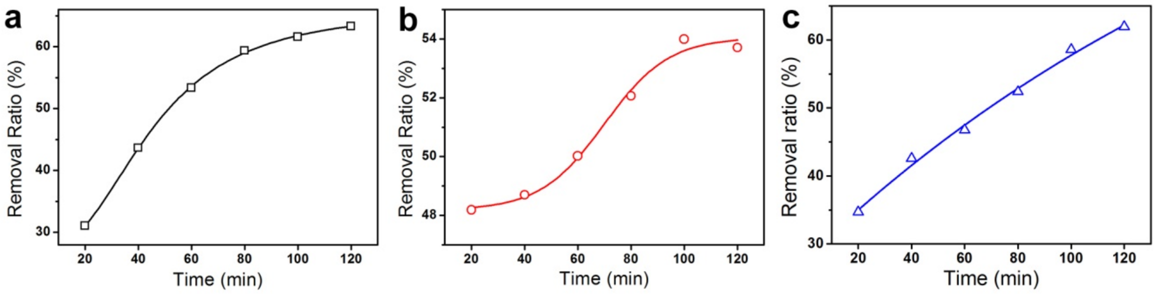

Figure S7. Removal ratio and pseudo-second-order kinetic models for US-N to mercury in removal 11 multiple metal ions. Removal ratio versus cycle time in (a) water, (b) artificial plasma, and (c) nature water. The curves in (a-c) are fitted.

3.4.3.2. Single Mercury Solution

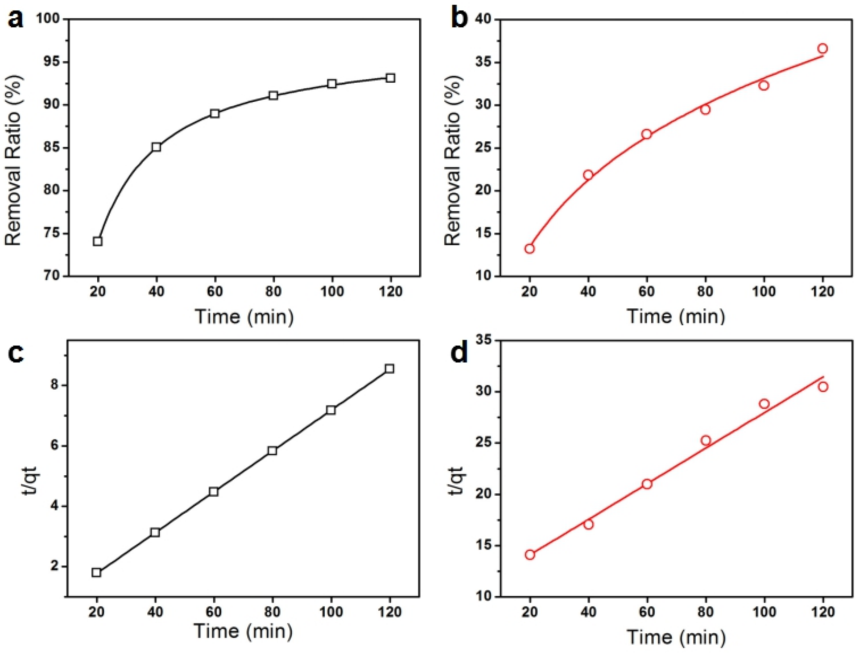

Figure S8. Removal ratio and pseudo-second-order kinetic models for US-N to mercury in single mercury removal. Removal ratio versus cycle time in (a) water, and (b) artificial plasma, pseudo-second-order kinetic models in (c) water, and (d) artificial plasma. The curves and lines in (a-d) are fitted.

3.5. Removal ratio of Hg under different concentration

Table S9. The removal ratio of Hg under different concentration.

Concentration of Hg solution (ppm)	Removal ratio of Hg
1	97.28%
2	96.10%
4	87.92%

3.6. Removal Kinetic Analysis

Table S10. The pseudo-second-order kinetic parameters for mercury removal in different situation.

Parameters	R-square	k_2	q_e	h
Water-Hg	0.99998	0.01073	14.7907	2.34797
Plasma-Hg	0.97356	0.00283	5.76136	0.09409
Water-Multiple	0.99456	0.00244	12.6294	0.38965
Plasma-Multiple	0.99633	0.03162	6.44122	1.31184
Nature-Multiple	0.97724	0.01586	2.13844	0.07252

3.7. Regeneration of US-N in mercury removal

After activation at 120°C for 2 h, 3 pieces of US-N membranes with 38 mm diameter were added to a circulation device (section 3.2) containing 200 ml of 1 ppm mercury solution, and the system was cycled for 2 h at room temperature. Finally, the residual metal content of the solution was measured by ICP-MS. After mercury adsorption, US-N membranes were immersed in 50 ml 0.1 M NH_4Cl solution for 2 h, then rinsed with deionized water, dehydrated with ethanol and dried at 60°C, and the US-N membranes were regenerated. And the regenerated US-N membranes were applied for mercury removal again.

Table S11. Mercury adsorption removal ratio after repeated regeneration.

Regeneration time	0	1	2	3	4	5	6	7	8	9
Removal ratio (%)	98.68	98.41	98.98	98.35	98.86	98.76	94.30	85.97	94.17	86.14

Reference

[1] S. Caroli, G. Forte, A.L. Iamiceli, A. Lusi, Stability of mercury dilute aqueous solutions: an open issue. *Microchem. J.* **1996**, 54, 418-428.

[2] J. Gao, Y. Zhang, W. Wei, et al. Liquid-infused micro-nanostructured MOF coatings (LIMNSMCs) with extremely high anti-icing performance. *ACS Applied Materials & Interfaces*, **2019**, 11, 47545-47552.

[3] J. Gao, M. Liu, W. Wei, et al. Preparation of large-size UiO-66-S films based on radiation grafted substrates and its mercury ion-removal performance in artificial plasma. *Journal of Radiation Research and Radiation Processing*, **2020**, 38, 29-38
